# Danazol mediates collateral sensitivity via STAT3/Myc related pathway in multidrug-resistant cancer cells

**DOI:** 10.1038/s41598-019-48169-2

**Published:** 2019-08-12

**Authors:** Ying-Tzu Chang, Yu-Ning Teng, Kun-I Lin, Charles C. N. Wang, Susan L. Morris-Natschke, Kuo-Hsiung Lee, Chin-Chuan Hung

**Affiliations:** 1 Department of Pharmacy, College of Pharmacy, China Medical University, 91 Hsueh-Shih Road, Taichung, 40402 Taiwan, ROC; 2 Department of Medicine, College of Medicine, I-Shou University, 8 Yida Road, Kaohsiung, 82445 Taiwan, ROC; 3 Department of Obstetrics and Gynecology, Chang Bing Show Chwan Memorial Hospital, Changhua, Taiwan, ROC; 4 Department of Cosmetic Science, Providence University, Taichung, Taiwan, ROC; 5 Department of Biomedical Informatics, Asia University, Wufeng, Taichung 41354 Taiwan, ROC; 6 Natural Products Research Laboratories, UNC Eshelman School of Pharmacy, University of North Carolina, Chapel Hill, North Carolina, 27599 United States; 7 Chinese Medicine Research and Development Center, China Medical University and Hospital, Taichung, 40447 Taiwan, ROC; 8 Department of Pharmacy, China Medical University Hospital, 2 Yude Road, Taichung, 40447 Taiwan, ROC

**Keywords:** Drug development, Cancer therapy

## Abstract

Multidrug resistance presents an obstacle in cancer treatment. Among numerous combative strategies, collateral sensitivity (CS) drugs have opened a new avenue to defeat cancer by exploiting selective toxicity against multidrug-resistant (MDR) cancer. In the present study, a clinically used synthetic steroid hormone, danazol, was investigated for its CS properties and cytotoxic mechanisms. Compared with natural hormones, danazol possessed a stronger selective cytotoxicity against MDR cancer cells. Danazol induced the arrest of MDR cancer cells at the G2/M phase and caspase-8–related early apoptosis. Furthermore, in MDR cancer cells, danazol reduced STAT3 phosphorylation as well as the expression of STAT3-regulated genes involved in cell survival, such as c-Myc, CDC25, and CDK1. Danazol also upregulated the cell cycle inhibitor p21 in MDR cancer cells. Supporting the experimental results, docking studies have revealed that danazol can likely bind favourably with STAT3. Taken together, our results suggest that danazol exerts a CS effect by inhibiting the STAT3 pathway in MDR cancer cells and thus provides a possible solution for MDR cancers.

## Introduction

Although several medical approaches, including immunotherapy (e.g., immune checkpoint inhibitors) and targeted therapy (e.g., tyrosine kinase inhibitors), have been added to cancer treatment, cytotoxic chemotherapeutic agents still play an crucial and first-line role in clinical practice^1,2^. However, because of multidrug resistance, patients do not enter remission, and tumours relapse and become insensitive to various structurally unrelated agents, leading to subsequent aggressive tumour growth or metastasis^1,2^. Previous studies have attributed the multidrug resistance phenomenon to several causes, including the overexpression of ATP-binding cassette (ABC) drug efflux transporters, genetic alteration during chemotherapy, rescue of the apoptosis signalling pathway, and damage repair mechanisms^3,4^.

Numerous approaches to combat multidrug-resistant (MDR) cancer have emerged in the past 50 years, such as developing new chemical structures that evade the efflux of ABC transporters, combining conventional chemotherapy with ABC transporter inhibitors, and exploiting MDR cancer cells based on distinct genetic properties compared with their chemosensitive counterparts^5^. Among the worldwide small-molecule drug screening projects, several research groups have reported that, unlike conventional chemotherapeutic agents that exhibit high resistance to MDR cancer cells, some drugs have the potential to target MDR cancer cells with a low response to sensitive cancer cells. This phenomenon is called collateral sensitivity (CS) and provides a prospective opportunity for combating MDR cancer^6,7^.

CS effects on cancer cell lines have been demonstrated for a wide range of drugs, including the L-type calcium channel antagonist verapamil^8^, flavonoid desmosdumotin B^9,10^, glucose analogue 2-deoxy-D-glucose (2-DG)^11^, iron chelator Dp44mT^12^, local anaesthetics and narcotics^13,14^, as well as some natural steroid hormones^15–17^. This novel MDR cancer-combating strategy has been related to several mechanisms, such as reactive oxygen species (ROS) production, energy utilisation sensitivity, extrusion of endogenous substrates required for cell survival, and membrane perturbation. However, results from some studies have been controversial^6^. In addition, the signal transducer and activator of transcription 3 (STAT3) signalling pathway has been associated with chemotherapeutic resistance in several cancers^18^. Silencing STAT3 may resensitise resistant cancer cells to paclitaxel, doxorubicin, and cisplatin^19^. More studies are warranted to elucidate the underlying mechanisms for the possible involvement of STAT3 in the CS effect.

**Figure 1:**
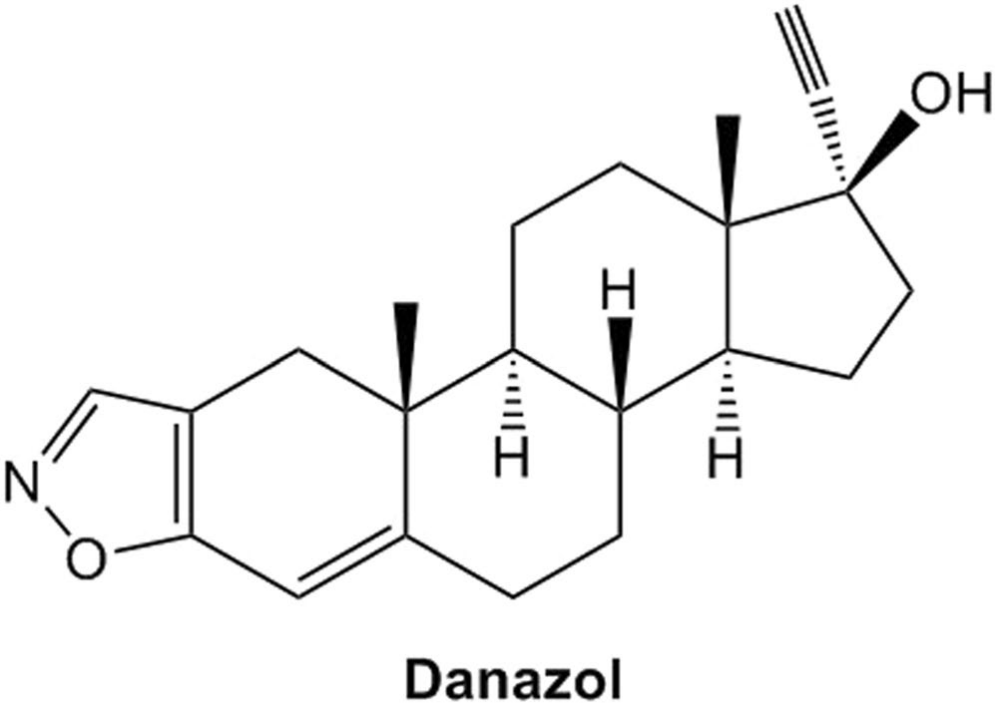
Structure of danazol.

Among the compounds that exhibited the CS effect, natural steroid hormones are the most promising candidates. Under physiological concentrations, progesterone and deoxycorticosterone demonstrated a considerable CS effect on MDR cancer cell lines^20^. Danazol (Fig. 1) is derived from testosterone and has been prescribed primarily for the treatment of endometriosis since 1971. In light of its diverse biological effects, danazol is also prescribed for ‘off-label’ use in a wide range of disorders^21,22^. Several studies have reported the cytotoxic effects of danazol alone and in combination with chemotherapeutic agents on leukaemia and breast cancer cells^23,24^. However, the CS effect of danazol has not been systemically evaluated. Therefore, our study was aimed at characterising the possible CS effect of danazol against MDR cancer cells and further elucidating the underlying mechanisms of CS to identify potential novel targets for future drug design.

## Results

### Danazol exhibited CS effect on several MDR cancer cell lines

Three paired parental (HeLaS3, HepG2, and NCI-H460) and MDR (KB/VIN, HepG2/VIN, and NCI-H460/MX20) cancer cell lines were used in SRB assay to screen both traditional chemotherapeutic agents and steroid hormones. From the results, MDR cancer cell lines exhibited strong resistance to chemotherapeutic agents: KB/VIN and HepG2/VIN were 3557.64- and 138.23-fold resistant toward paclitaxel and NCI-H460/MX20 was 51.32- and 39.72- fold resistant toward mitoxantrone and etoposide, respectively (Table 1).

**Table 1:** Selectivity Index of Danazol on several multidrug-resistant cancer cell lines.

Compound	IC_50_		Resistant Fold (RF)^a^	Selectivity Index (SI)^b^
	HeLaS3	KB/VIN		
Danazol (μM)	24.44 ± 1.87	0.73 ± 0.04		33.48
Doxorubicin (nM)	503.74 ± 63.65	12156.70 ± 1.00	24.13	
Paclitaxel (nM)	0.78 ± 0.02	2774.96 ± 71.56	3557.64	
	**HepG2**	**HepG2/VIN**		
Danazol (μM)	25.42 ± 3.48	0.12 ± 0.01		211.83
Paclitaxel (nM)	14.27 ± 2.46	1972.55 ± 23.50	138.23	
Vincristine (nM)	75.68 ± 4.87	2457.60 ± 113.96	32.47	
	**NCI-H460**	**NCI-H460/MX20**		
Danazol (μM)	79.55 ± 5.05	17.89 ± 0.80		4.45
Doxorubicin (nM)	80.59 ± 2.46	1944.14 ± 41.93	24.12	
Etoposide (nM)	666.32 ± 16.90	26464.05 ± 3692	39.72	
Mitoxantrone (nM)	81.22 ± 3.81	4168.22 ± 165.84	51.32	
Topotecan (nM)	55.33 ± 3.18	1437.22 ± 152.95	25.98	

^a^The resistant fold was calculated by dividing the individual IC_50_ of MDR cell line (KB/VIN, HepG2/VIN, or NCI-H460/MX20) by the IC_50_ of parental cell line (HeLaS3, HepG2, or NCI-H460).

^b^The selectivity index was calculated by dividing the individual IC_50_ of parental cell line (HeLaS3, HepG2, or NCI-H460) by the IC_50_ of MDR cell line (KB/VIN, HepG2/VIN, or NCI-H460/MX20).

Both natural and synthetic hormones were tested in our primary screening for their CS effects on KB/VIN. The results in Table 2 indicated that the synthetic steroid hormone danazol exhibited the most potent cytotoxicity (0.73 μM) against KB/VIN among the screened compounds (selectivity index (SI) was 33.48). Some natural steroid hormones also showed different degrees of cytotoxic selectivity toward KB/VIN, while aldosterone and estriol exhibited no difference toward parental and resistant cell lines. Notably, the order of the SI of pregnenolone, progesterone, deoxycorticosterone, and corticosterone corresponded generally to the order of drug metabolism. The selectivity gradually increased when pregnenolone was metabolized to corticosterone (SI 2.68, 5.21, 8.99 and 7.81).

**Table 2:** Cytotoxicity of natural and synthetic steroid hormones on HeLaS3 (parental) and KB/VIN (MDR) cancer cell lines.

Compound	IC_50_		Resistant Fold (RF)^a^	Selectivity Index (SI)^b^
	HeLaS3	KB/VIN		
**Natural and synthetic steroid hormone**				
Aldosterone (μM)	>40	>40		N/A^c^
β-estradiol (μM)	19.52 ± 0.52	7.02 ± 0.18		2.78
Corticosterone (μM)	197.94 ± 4.79	25.36 ± 5.70		7.81
Danazol (μM)	24.44 ± 1.87	0.73 ± 0.04		33.48
Deoxycorticosterone (μM)	55.46 ± 3.91	6.17 ± 0.59		8.99
Estriol (μM)	38.98 ± 1.77	49.99 ± 1.55		0.78
Pregneolone (μM)	40.66 ± 1.81	15.20 ± 0.69		2.68
Progesterone (μM)	32.42 ± 0.22	6.22 ± 0.56		5.21
**Chemotherapeutic agent**				
Doxorubicin (nM)	503.74 ± 63.65	12156.70 ± 1.00	24.13	
Paclitaxel (nM)	0.78 ± 0.02	2774.96 ± 71.56	3557.64	

^a^The resistant fold was calculated by dividing the individual IC_50_ of KB/VIN by the IC_50_ of HeLaS3.

^b^The selectivity index was calculated by dividing the individual IC_50_ of HeLaS3 by the IC_50_ of KB/VIN.

^c^Not available.

Danazol, the most potent CS compound, was further investigated for selectivity toward other MDR cancer cell lines. It also exhibited strong and moderate selectivity toward HepG2/VIN (SI 211.83) and NCI-H460/MX20 (SI 4.45), demonstrating CS effects across multiple kinds of MDR cancer cell lines (Table 1).

### The CS effect of danazol was not related to the efflux function of P-glycoprotein

**Figure 2:**
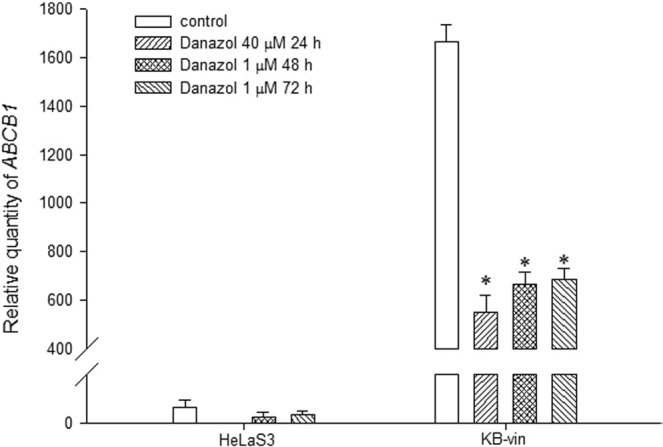
Results of *ABCB1* gene expression analysis. HeLaS3 and KB/VIN were treated with danazol for 24, 48, and 72 hours. Total RNA was extracted and *ABCB1* gene expression level of each sample was quantified by real-time PCR. The *ABCB1* gene expression was significantly down-regulated by danazol treatment in KB/VIN cell line. Statistical differences were evaluated by ANOVA followed post hoc analysis (Tukey’s test). * Indicates p value < 0.05 compared with control group. Data presented as mean ± SE of at least three experiments, each in triplicate.

The CS mechanism is often linked to the drug efflux pump P-glycoprotein (P-gp), which is responsible for one mechanism leading to the MDR cancer phenotype. Therefore, the relationship between the CS effect of danazol and the expression and function of P-gp was investigated. Danazol was co-administered with a P-gp substrate or inhibitor to evaluate whether the P-gp efflux function influenced the compound’s CS effect. The cytotoxic IC_50_ of danazol was not significantly altered after co-treatment with the P-gp substrate vincristine or the P-gp inhibitors verapamil and cyclosporin A. The IC_50_ values for danazol only, danazol with 100 nM vincristine, danazol with 10 μM verapamil, and danazol with 1 μM cyclosporin A in KB/VIN cells were 0.73 ± 0.04, 0.66 ± 0.02, 0.46 ± 0.11, and 0.49 ± 0.13 μM, respectively. Furthermore, the IC_50_ values of danazol in the human P-gp stable expression cell line *ABCB1*/ Flp-In^TM^-293 and the parental cell Flp-In^TM^-293 were similar (13.76 ± 0.13 and 13.52 ± 0.54 μM). Although treatment with danazol significantly down-regulated the *ABCB1* mRNA expression level in KB/VIN (Fig. 2), the compound’s SI was not correlated to P-gp expression level and function.

### Danazol arrested cell cycle at G2/M phase and induced apoptosis in MDR KB/VIN cell line

**Figure 3:**
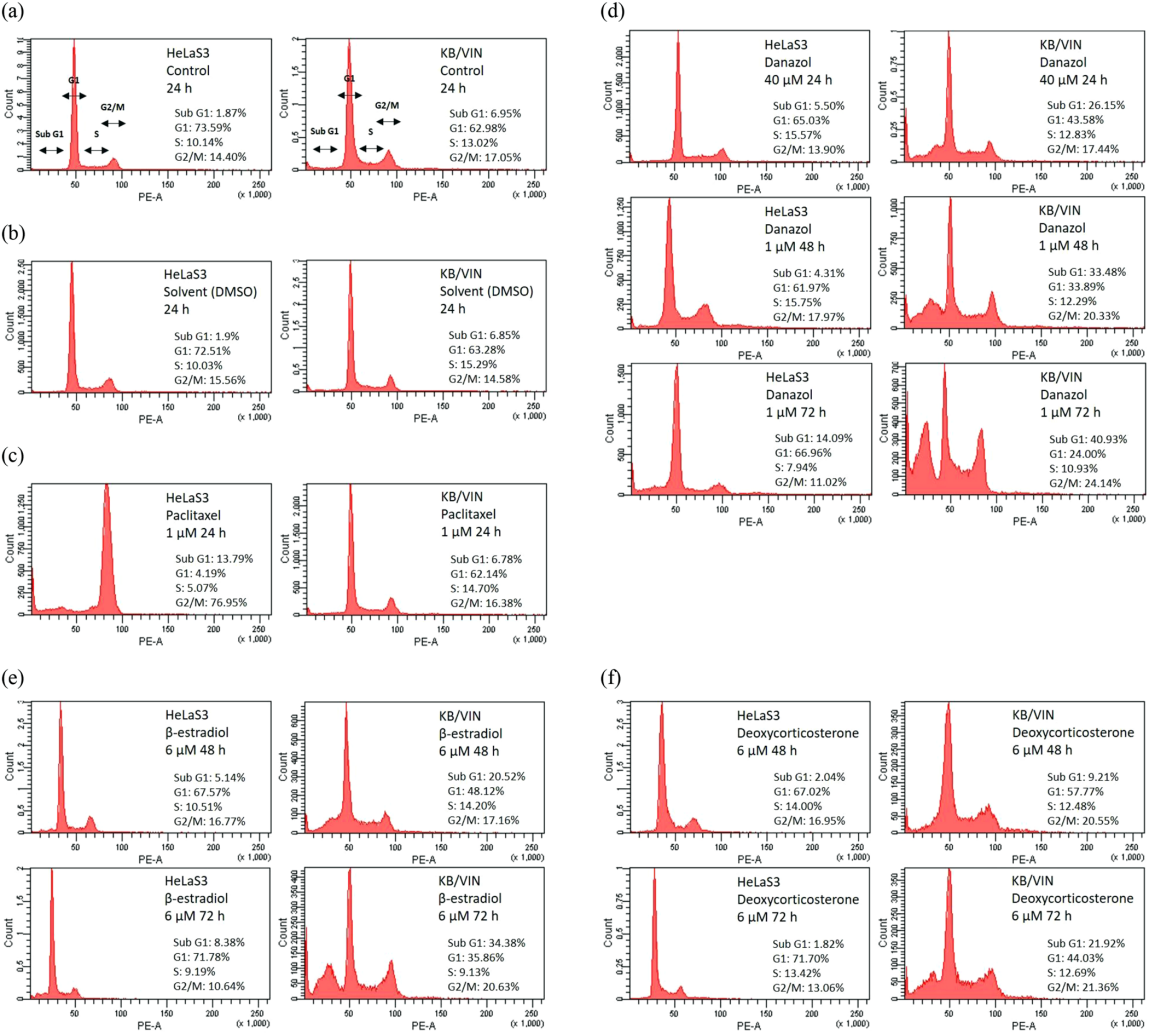
Results of cell cycle analysis in HeLaS3 and MDR KB/VIN cells. HeLaS3 and KB/VIN were treated with culture medium (**a**), DMSO (**b**), paclitaxel (**c**; positive control), danazol (**d**), β-estradiol (**e**), and deoxycorticosterone (**f**) for 24, 48, and 72 hours. DNA contents and cell cycle distribution of each sample were determined by PI solution (X-axis PE). Danazol, β-estradiol, and deoxycorticosterone arrested KB/VIN cell at G2/M phase and caused apoptosis (increased sub G1) in a time-dependent manner.

**Figure 4:**
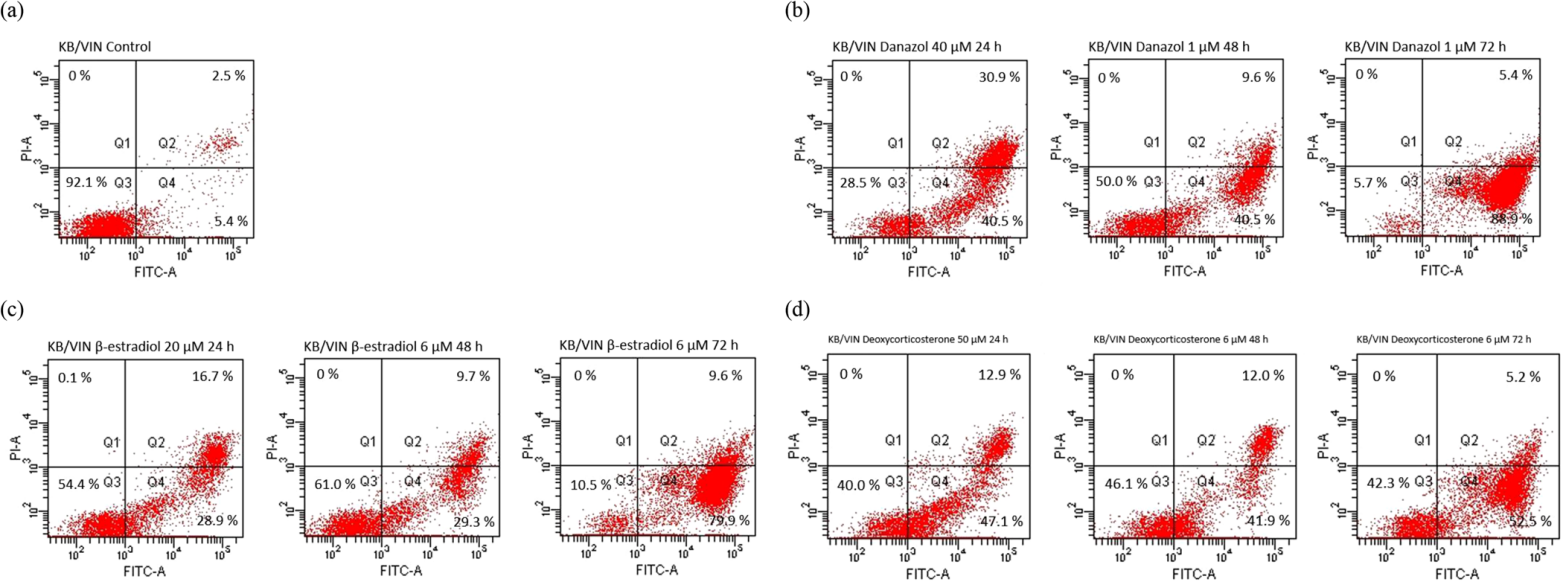
Results of apoptosis assay in MDR KB/VIN cells. KB/VIN were treated with culture medium (**a**), danazol (**b**), β-estradiol (**c**), and deoxycorticosterone (**d**) for 24, 48, and 72 hours. Apoptosis and necrosis status of each sample was determined by annexin V (X-axis FITC) and PI (Y-axis PI). Cells distributed in Q1, Q2, Q3, and Q4 represented necrosis, late-apoptosis, normal, and early-apoptosis, respectively. Danazol, β-estradiol, and deoxycorticosterone exhibited prominent cell early-apoptosis after 72 hours treatment.

Based on the cytotoxicity data, danazol and two natural steroid hormones (β-estradiol and deoxycorticosterone) were selected for cell cycle and apoptosis analysis. β-Estradiol and deoxycorticosterone represent different cholesterol metabolic pathways and, thus, both were used for comparison purposes. The results are shown in Figs 3 and 4. In the cell cycle distribution analysis, we first treated 40x higher concentration of danazol for 24 h to evaluate the acute selective-effect of danazol in MDR cell lines. The results showed that KB/VIN cells represented an increase in the subG1-phase after high-dose and short-term danazol treatment. On the other hand, KB/VIN cells exhibited normal cell cycle distribution after treatment of 1 μM paclitaxel while parental HeLaS3 cells did not, demonstrating the resistance of KB/VIN cells to paclitaxel (Fig. 3c). The long-term cytotoxic effect was further evaluated by 48 h and 72 h treatments. Danazol, β-estradiol, and deoxycorticosterone all arrested KB/VIN cells at the G2/M phase in a time-dependent manner, while the cell cycle distribution in HeLaS3 remained the same as control regardless of the treatment dosages and time (Fig. 3d–f).

Results in the apoptosis assay revealed that danazol, β-estradiol, and deoxycorticosterone elicited significant early-apoptosis after 72 h treatment in the KB/VIN cell line (Fig. 4b–d). These results were consistent with the cell cycle analysis data, demonstrating that the cytotoxicity of steroid hormones on the MDR cell line KB/VIN resulted from cell apoptosis and was cell cycle dependent.

### Danazol modulated apoptosis in KB/VIN cells through ROS and caspase-8 activation

**Figure 5:**
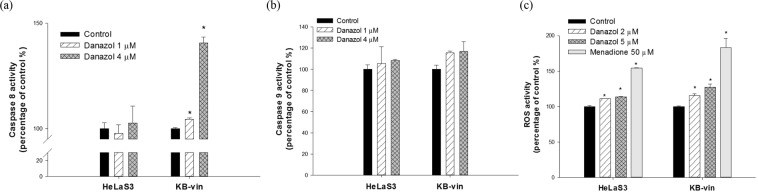
Results of caspase activity and ROS levels detection assay in HeLaS3 and MDR KB/VIN cells. HeLaS3 and KB/VIN cells were treated with or without danazol for 72 hours. The activities of caspase-8 (**a**) and caspase-9 (**b**) were evaluated by Cell Meter™ Caspase Activity Apoptosis Assay Kits. Danazol activated caspase-8 in KB/VIN cells while the level remained the same in HeLaS3 cells compared with respective control group. (**c**) The treatment of danazol increased the ROS level in both HeLaS3 and KB/VIN cell lines. Menadione, a ROS inducer, was used as a positive control. Statistical differences were evaluated by the ANOVA followed post hoc analysis (Tukey’s test). * indicates p value < 0.05 compared with control group. Data presented as mean ± SE of at least two experiments, each in duplicate.

To clarify whether the apoptosis induced by danazol was related to caspase activation or ROS-induction, a caspase activity detection assay was performed with Cell Meter™ apoptosis assay kits for caspases 8 and 9 activity. Danazol significantly activated caspase-8 in the KB/VIN, but not the HeLaS33, cell line (Fig. 5a). No significant effect was seen on caspase-9 activity (Fig. 5b). Moreover, danazol elicited high ROS levels in HeLaS3 as well as KB/VIN cells (Fig. 5c). These results demonstrated the compound’s selective property and apoptosis regulation in MDR cancer cells.

### Danazol arrested G2/M cell cycle in KB/VIN cells by suppressing STAT3

**Figure 6:**
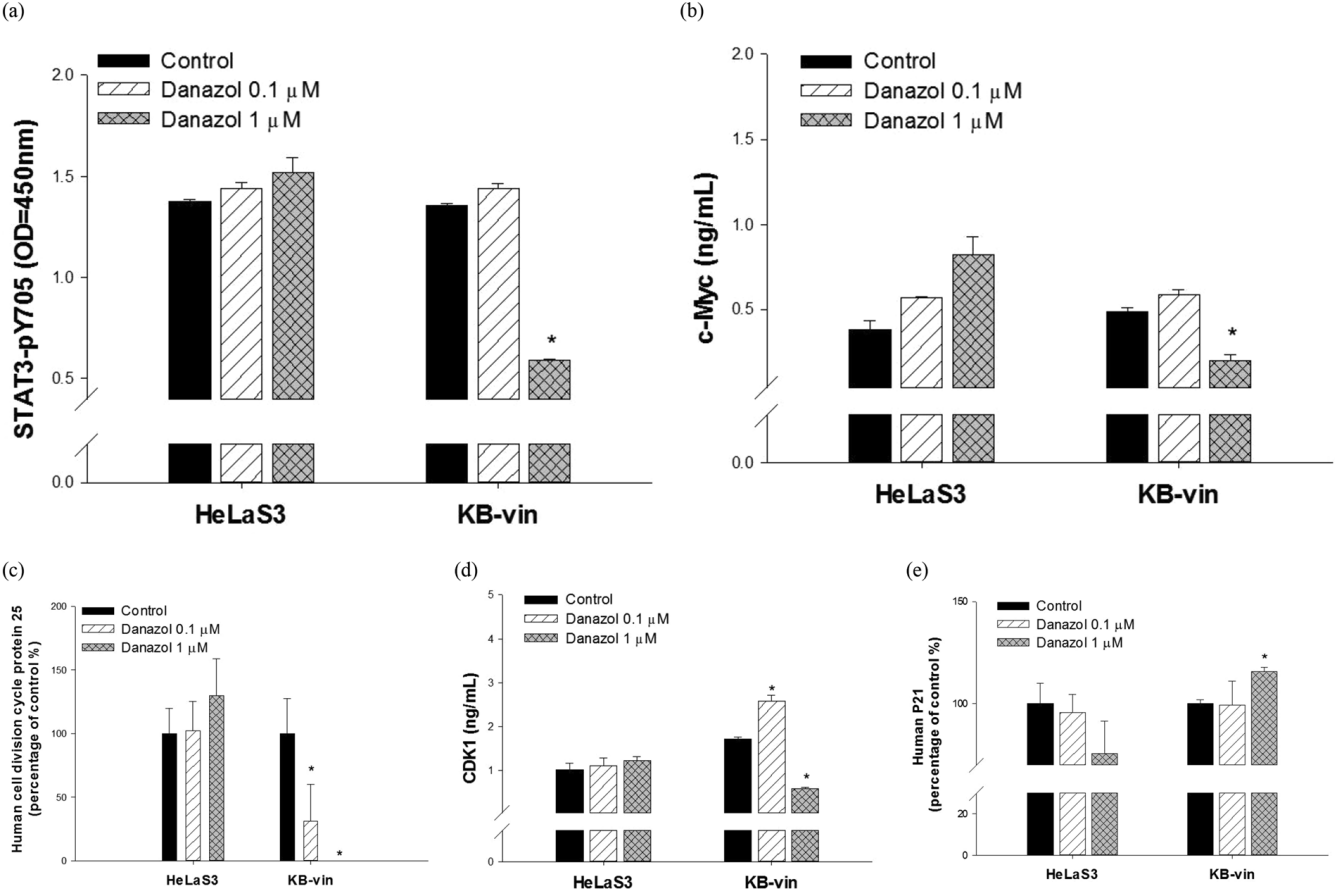
MYC signaling mediates danazol-induced G2/M arrest in MDR KB/VIN cells. HeLaS3 and KB/VIN were treated with or without danazol for 72 hours. The whole-treated cells were extracted and analyzed by ELISA using antibodies specific to human (**a**) STAT3-pY705, (**b**) cMYC, (**c**) CDC25, (**d**) CDK1, and (**e**) p21. The results showed danazol reduced the concentration of MYC (c-Myc) and its upstream protein STAT3-pY705 in MDR KB/VIN cells. In addition, decreases in downstream signaling proteins, CDC25 and CDK1, were shown. Each experiment was performed in duplicate. Student’s t-test, *p < 0.05 compared with cells treated with medium only control.

We further investigated the effects of danazol on STAT3-related pathways. ELISA was performed to reveal alterations of the target protein after danazol treatment. The concentrations of danazol were the same as the 72 h treatment of apoptosis assay and cell cycle analysis. Our data showed that danazol significantly reduced the phosphorylation of STAT3 as well as downstream MYC expression in MDR KB/VIN cells but not the parental HeLaS3 cells (Fig. 6a,b). In addition, the ELISA results demonstrated that danazol treatment inhibited CDC25 and CDK1 activation in KB/VIN cells (Fig. 6c,d). Furthermore, danazol treatment also resulted in increased levels of p21 in KB/VIN cells (Fig. 6e).

### Docking simulation

**Figure 7:**
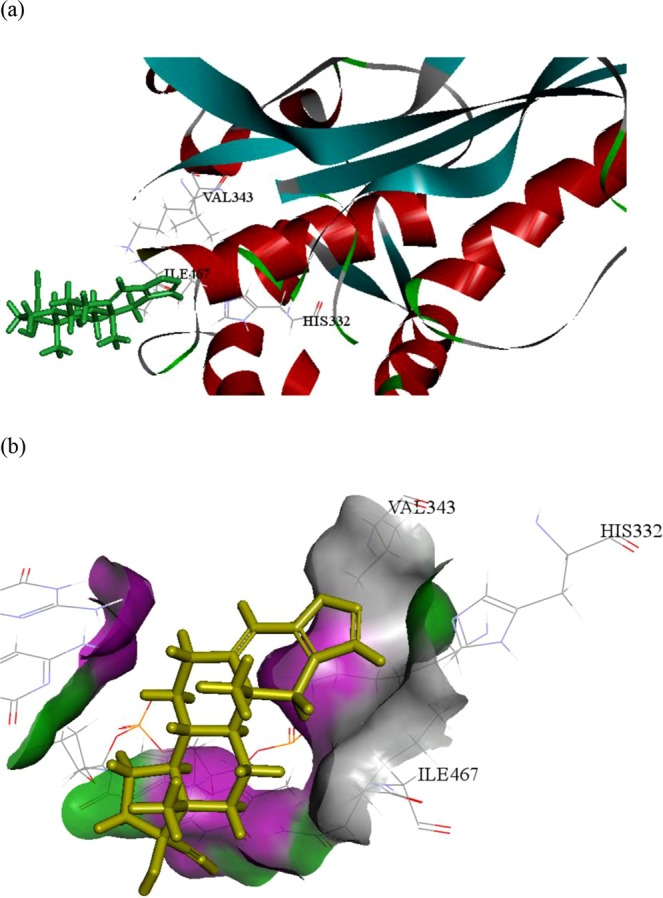
Molecular docking analysis of danazol with STAT3. (**a**) Superimposition of docked position of compound in the STAT3-binding pocket of 3D structure (PDB entry 1BG1). (**b**) 3D model representation of the adduct danazol with STAT3.

In order to investigate the supposed binding pattern and possible interaction between danazol and the STAT3 ligand pocket, danazol was virtually docked with crystal structures of protein’s ligand-binding domain (PDB entry 1BG1) using the docking program C-DOCKER. The best docking results of danazol with the active site of STAT3 provided a C-DOCKER energy score of 40.2737 and binding energy of 46.0273 Kcal/mol. The binding model clearly indicated that danazol interacts with certain STAT3 residues, such as HIS332, ILE467 and VAL343 (Fig. 7).

## Discussion

The recurrent occurrence of multidrug resistance in cancer treatment has stimulated research and development on multidrug resistance-reversing agents and selectively cytotoxic agents. In the present study, we observed that a synthetic hormone steroid and clinically used drug, danazol, exhibited CS towards several MDR cancer cell lines (KB/VIN, HepG2/VIN, and NCI-H460/MX20). Danazol induced KB/VIN death through cell cycle G2/M arrest and caspase-8 activation. Furthermore, danazol may mediate the CS effect through the STAT3/MYC pathway.

Based on previous research efforts, four possible cytotoxic mechanisms have been linked to CS in MDR cancer cell lines. The first mechanism is ROS overproduction induced by CS compounds. The two most well-known examples are verapamil^25^ and NSC73306^26^, which represent the P-gp–related and redox cycling-related ROS generation, respectively. Compared with parental cells, MDR cancer cells can more easily reach high ROS levels and initiate apoptosis. Second, MDR cancer cells are more sensitive to energy level changes. As a result, compounds that interfere with certain cellular metabolic pathways or the electron transport chain, such as 2-DG and rotenone, have demonstrated the CS effect^27^. The third mechanism is also related to ABC transporters. This hypothesis proposes that CS agents do not directly mediate MDR cytotoxicity but instead affect other major life-related endogenous substances, which are pumped out by ABC transporters as facilitated by the CS agents. Therefore, the compounds influence cytotoxicity indirectly^28^. The final theory is related to the membrane structure. The membranes of MDR cancer cells that harbour abundant P-gp are prone to changes in structure and fluidity, which can be caused by some CS agents, resulting in the nonintact cell phenotype and cell death^14^. Because CS agents induce cell death through various mechanisms, they need to be analysed on a drug-by-drug basis. In the present study, we discovered that the CS effect of danazol was not related to P-gp. Alternatively, the study revealed that danazol can act on the STAT3/cMYC pathway and may regulate MDR cell apoptosis through caspase-8 activation. Furthermore, our molecular docking study demonstrated that danazol could have direct interactions with STAT3 and cMYC. This result provided additional evidence that danazol may mediate the CS effect by regulating the STAT3/cMYC pathway.

Several steroid hormones, including 5β-pregnane-3,20-dione, deoxycorticosterone^13^, prednisolone^15^, dexamethasone^16^, progesterone^20^, and megestrol acetate^17^, have demonstrated the CS effect against MDR cancer cells. Representative natural steroid hormones from various subcategories (e.g., progestogens and glucocorticoids) and points on the cholesterol metabolic pathway of natural steroids were evaluated in our primary cytotoxicity screening. An increased cytotoxic selectivity towards KB/VIN was observed along the pregnenolone, progesterone, deoxycorticosterone, and corticosterone metabolic pathways. These findings may be helpful for the design of future CS agents.

STAT3 belongs to the signal transducer and activator of transcription family, and phosphorylated STAT3 is involved in cancer proliferation, invasion, and metastasis through its modulation of the transcription of genes related to cell survival^29,30^. Several studies have indicated that inhibition of the STAT3 pathway leads to the inhibition of tumour growth both *in vivo* and *in vitro*^31^. Furthermore, STAT3 upregulation may be associated with drug resistance in cancer treatment^32–34^. Attenuation of the STAT3 signalling pathway resensitised resistant cancer cells to chemotherapeutic agents as well as inhibited cancer growth^34,35^. We observed that danazol decreased STAT3 phosphorylation, and the correlated results from an *in silico* docking simulation also suggested that danazol could be a good inhibitor of STAT3.

In conclusion, the present study demonstrated that danazol is a potent CS agent. Because it is used prevalently with known safety data in clinical applications, danazol may provide a novel choice in future MDR cancer treatment strategies. The novel regulating pathway ascertained in this research and the scaffold of danazol could be applied as a molecular target and compound lead, respectively, in the development of more effective multidrug resistance-selective cytotoxic agents.

## Materials and Methods

### Chemicals and reagents

Acetic acid, aldosterone, β-estradiol, corticosterone, cyclosporin A, danazol, deoxycorticosterone, dimethyl sulfoxide, doxorubicin, estriol, etoposide, mitoxantrone, paclitaxel, pregnenolone, progesterone, menadione, sulforhodamine B (SRB), trichloroacetic acid (TCA), topotecan, tris base, verapamil, and vincristine were purchased from Sigma Chemical Co (St. Louis, MO, USA). DMEM and RPMI-1640 media, phosphate buffered saline (PBS), penicillin–streptomycin, fetal bovine serum (FBS), and trypsin–EDTA were purchased from Thermo Fisher Scientific Inc. All steroids and chemotherapeutic drugs were dissolved in 0.1% DMSO, which was used as the control treatment in all experiments.

### Parental and multi-drug resistant cancer cell lines

Parental cancer cell lines, including human cervical epithelioid carcinoma HeLaS3, human hepatoblastoma HepG2, and human non-small cell lung carcinoma NCI-H460, were purchased from Bioresource Collection and Research Center (Hsinchu, Taiwan). The multi-drug resistant human cervical cancer cell line KB/VIN was kindly provided by Dr. Kuo-Hsiung Lee (University of North Carolina, Chapel Hill, U.S.A). The drug resistance property of KB/VIN was induced with gradually increased concentrations of vincristine and maintained regularly with vincristine. The other MDR cancer cell lines were generated as we previously reported^36^. Briefly, the parental cancer cell lines were treated with gradual concentrations of chemotherapeutic agents from the 72 h IC_50_. After overnight treatment, the cells were incubated in drug-free medium for 3 days. Repeating above process and dose titration for each treatment until the acquired resistance cell lines reach 100-fold less sensitive to chemotherapeutic agents compared with the parental cell lines. The multi-drug resistant human hepatocellular carcinoma cell line HepG2/VIN was selected from HepG2 with gradually increased concentrations of vincristine. The multi-drug resistant human non-small cell lung carcinoma cell line NCI-H460/MX20 was selected from NCI-H460 with gradually increased concentrations of mitoxantrone. All cells were cultured in RPMI-1640 containing 10% FBS at 37 °C in a humidified atmosphere of 5% CO_2_.

### Human P-gp stable expression cell line (*ABCB1*/ Flp-In^TM^-293)

The human P-gp stable expression cell line was constructed previously^37^. Briefly, the constructed *ABCB1*/pcDNA5 was co-transfected with pOG44 (the Flp recombinase expression plasmid) into the Flp-In^TM^-293 cells. A stable clone cell line was selected based on hygromycin B resistance, while parental Flp-In^TM^-293 cells were selected by zeocin resistance. All cells were cultured in Dulbecco’s modified eagle’s medium supplemented with 10% FBS at 37 °C, 95% humidity, and 5% CO_2_. The protein expression and function of P-gp were confirmed in our previous work^38^.

### Real-time quantitative RT-PCR

*ABCB1* mRNA expression levels were quantified by real-time RT-PCR. Total RNA was extracted from transfected cells using Qiagen RNeasy kit (Valencia, CA, USA). Taqman Assay-on-Demand reagents (primers and probe sets) for *ABCB1* (Hs00184500_m1) and *GAPDH* (Hs02758991_g1) genes were provided by Applied Biosystem (Foster City, CA, USA). The relative *ABCB1* mRNA expression levels were normalized to the amount of *GAPDH* in the same cDNA and evaluated by ABI Prism 7900 Sequence Detection System.

### Cytotoxicity assay and the calculation of resistant fold and selectivity index

The cytotoxicity of the hormone steroids was evaluated by the sulforhodamine B (SRB) assay through determination of total protein amounts. Briefly, after 72 h treatment with drugs, 50% trichloroacetic acid (TCA) was added for 30 min to fix cells, which then were washed with water and air-dried. Afterward, cells were stained with 0.04% SRB for 30 min and then washed with 1% acetic acid to remove unbound dye and air-dried. The bound stain was solubilized in 10 mM Tris Base and the absorbance was measured on a BioTek Synergy HT Multi-Mode Microplate Reader at 515 nm. Three dose response parameters were calculated for each experimental agent. Growth inhibition of 50% (IC_50_) was calculated from [(Ti − Tz)/(C − Tz)] × 100 = 50 (Tz: time zero; C: control growth; Ti: growth of tested group in the presence of drug at the different concentration levels), which is the drug concentration resulting in a 50% reduction of the net protein increase (as measured by SRB staining) in control cells during the drug incubation.

The Resistance Fold (RF) was calculated by dividing the individual IC_50_ of MDR cell line (KB/VIN, HepG2/VIN, or NCI-H460/MX20) by the IC_50_ of parental cell line (HeLaS3, HepG2, or NCI-H460). The Selectivity Index (SI) was calculated by dividing the individual IC_50_ of parental cell line (HeLaS3, HepG2, or NCI-H460) by the IC_50_ of MDR cell line (KB/VIN, HepG2/VIN, or NCI-H460/MX20). A higher RF value indicates that the MDR cancer cells showed higher resistance to the compound, while a higher SI value indicates that the MDR cancer cells showed higher response to the compound.

### Cell cycle analysis

HeLaS3 and KB/VIN cells were plated on 6-well plates with serum-free medium for starvation. After 24 h, cells were treated with β-estradiol, danazol, or deoxycorticosterone. We assessed the acute cytotoxicity by high-dose (40x higher concentration) and short-term (24 h) of danazol treatment, and the long-term effect was evaluated by 48 h and 72 h treatments. After different time periods, cells were harvested and washed in cold phosphate-buffered saline (PBS), followed by fixing in ice-cold 70% ethanol for at least 24 h. Then, cells were incubated with 50 μg/mL PI at 4 °C for 24 h in the dark. Cells were then analyzed by FACS analysis (BD FACSCanto System with excitation laser 488 nm, measuring emission at 575 nm for PI).

### Apoptosis assay

Apoptosis evaluations were performed with an Alexa Fluor^®^ 488 annexin V/Dead Cell Apoptosis Kit from Molecular Probes^®^ (Cat. No. V13241). KB/VIN cells were plated on 6-well plates. After 24 h, cells were treated with β-estradiol, danazol, or deoxycorticosterone. At different time points, cells were harvested, washed in cold phosphate-buffered saline (PBS), and then re-suspended in 1X annexin-binding buffer. For each assay, 100 μL of re-suspended cells (100 μL) were adequate (about 1 × 10^6^ cells/mL). Alexa Fluor^®^ 488 annexin V (5 μL) and 1 μL 100 μg/mL PI working solution (1 μL) were added to each 100 μL cell suspension and then cells were incubated at room temperature for 15 min. After the incubation period, 400 μL of 1X annexin-binding buffer were added to each sample and the stained cells were then analyzed by FACS analysis (BD FACSCanto System with excitation laser 488 nm, measuring emission at 530 nm for FITC and 575 nm for PI).

### Caspase activity detection assay

Cell Meter™ Apoptosis Assay Kits for caspases 8 and 9 activity (AAT Bioquest®, Inc.) were used for caspase activity evaluation. HeLaS3 and KB/VIN cells were seeded on 96-well black plates and treated with 1 and 4 folds of 72 h IC_50_ of danazol for 4 h to assess the dose-dependent effect according the instruction manual. After the incubation, 100 µL/well assay loading solution was added to the plates, which were then incubated in the dark for 1. Then, the fluorescence was measured with a BioTek Synergy HT Multi-Mode Microplate Reader at Ex/Em = 490/525 nm.

### Reactive oxygen species (ROS) detection assay

The intracellular levels of ROS were measured with a Cell Meter™ Fluorimetric Intracellular Total ROS Activity Assay Kit (AAT Bioquest®, Inc.). HeLaS3 and KB/VIN cells were seeded on 96-well black plates and incubated overnight. The next day, 100 µL/well of Amplite™ ROS Green working solution was added for 1 h, then 2 and 5 folds of 72 h IC_50_ of danazol was added for 2 h to assess the ROS inducing and dose-dependent effect. The fluorescence was measured by using a BioTek Synergy HT Multi-Mode Microplate Reader at Ex/Em = 490/525 nm.

### Enzyme-linked immunosorbent assay (ELISA)

The levels of CDK1, MYC, p21, CDC25, and STAT3-pY705 were measured by standard ELISA according to manufacturer’s instructions (Elabscience®, Catalog No: E-EL-H2321, E-EL-H0756, and E-EL-H1740; Mybiosource, Catalog No: MBS452073; Abcam®, Catalog No: ab126458). Briefly, HeLaS3 and KB/VIN cells were seeded onto 6-well plates and treated with danazol for 72 h. After the incubation, cells were harvested and total cellular proteins were extracted for further experiments.

### Molecular docking

The docking software CDOCKER with BIOVIA Discovery Studio 4.5 (D.S. 4.5) were used for the docking simulation. CDOCKER generate random conformations by using the CHARMm-based docking engine to perform flexible ligand-based docking and docking refinement. The crystal structure of STAT3 (PDB ID: 1BG1)^39^ was obtained from the RCSB Protein Data Bank. The structure was selected based on the following criteria: (1) a crystal structure with a resolution lower than 2.25 Å, (2) a crystal structure of the STAT3 homodimer bound to DNA, and (3) a crystal structure with no known mutation. The binding site was prepared using the location of the co-crystallized ligand. Docking was performed using the CDOCKER docking module of DS 4.5. The docking strategy used the hybrid (enthalpy and entropy) approach. For each complex, 1000 docking simulations were performed with default parameters.

### Statistical analysis

Statistical differences were evaluated by ANOVA followed post hoc analysis (Tukey’s test) or the Student’s t-test. The statistical significance was set at p value < 0.05.
